# Cognitive and Affective Dysregulation in Neuropathic Pain: Associated Hippocampal Remodeling and Microglial Activation

**DOI:** 10.3390/ijms26136460

**Published:** 2025-07-04

**Authors:** Anna Tyrtyshnaia, Igor Manzhulo, Anastasia Egoraeva, Darya Ivashkevich

**Affiliations:** A.V. Zhirmunsky National Scientific Center of Marine Biology, Far Eastern Branch, Russian Academy of Sciences, 690041 Vladivostok, Russia; i-manzhulo@bk.ru (I.M.); egoraeva_a@bk.ru (A.E.); owncean@yandex.ru (D.I.)

**Keywords:** neuropathic pain, sparing nerve injury, hippocampus, working memory, long-term memory, neurogenesis, microglia, astroglia

## Abstract

Neuropathic pain is a persistent and exhausting condition which results from damage to the nervous system and is often accompanied by emotional and cognitive impairments. In this study, we investigated dynamic changes in pain-related behaviors over 8 weeks using a spared nerve injury (SNI) model in male C57Bl/6 mice. We examined behavioral outcomes in conjunction with glial activation, neurogenesis, and glutamatergic signaling in the hippocampus to elucidate the mechanisms underlying cognitive and affective alterations associated with chronic pain. Our findings demonstrate that SNI-induced neuropathic pain progressively increases anxiety-like behavior and impairs both working and long-term memory. These behavioral deficits are accompanied by significant activation of microglia and astrocytes, a reduction in hippocampal neurogenesis, and a decrease in the expression of NMDA and AMPA glutamate receptor subunits and the scaffolding protein PSD-95. Taken together, our results suggest that hippocampal neuroinflammation and associated synaptic dysfunction contribute to the affective and cognitive disturbances observed in chronic pain, providing insight into potential molecular targets for therapeutic intervention.

## 1. Introduction

Neuropathic pain is an exhausting condition characterized not only by intense, chronic pain but also by significant behavioral and cognitive alterations, which are increasingly recognized to be linked to underlying changes in the central nervous system [1].

Recent epidemiological studies estimate the global prevalence of chronic neuropathic pain to be 7–13% in the general population, with rates exceeding 20% in individuals with diabetes, post-surgical syndromes, or cancer [2,3]. These findings underscore the importance of mechanistic studies targeting central maladaptive changes.

Changes involved in neuropathic pain include microglial activation, impaired neurogenesis, and alterations in the neurotransmission and expression of glutamate receptors and synaptic proteins [4,5]. Studying behavioral changes and hippocampal neuroplasticity in neuropathic pain is critical because the hippocampus, a brain region crucial for memory and learning, is increasingly implicated in the development of chronic pain, particularly the emotional and cognitive aspects associated with it. This means that alterations in hippocampal function can contribute significantly to the behavioral changes seen in people experiencing neuropathic pain, like anxiety and depression, and provide insights into potential therapeutic targets for managing these symptoms [1].

Changes in microglia and astroglia activation, particularly in the hippocampus, along with alterations in glutamate receptor expression, are strongly linked to the development of neuropathic pain. Such changes can contribute to cognitive and behavioral disorders by disrupting the delicate balance of neurotransmission and synaptic plasticity in the brain, primarily through inflammatory signaling and altered neuronal activity [6].

When a peripheral nerve is damaged, microglia in the spinal cord and brain regions like the hippocampus become activated, releasing pro-inflammatory cytokines (e.g., TNF-alpha, IL-1beta) which can further stimulate neuronal excitability, leading to perception of pain and disruption of cognitive function [7]. Activated microglia can also influence astrocytes, causing them to lose their ability to effectively clear glutamate from the synaptic cleft, leading to excessive glutamate signaling and neuronal hyperexcitability, further contributing to pain and cognitive impairment [8]. Changes in the expression and function of glutamate receptors, particularly NMDA receptors, on neurons can occur due to glial activation. This can lead to increased neuronal firing and contribute to central sensitization, a key mechanism in neuropathic pain [9]. Activated microglia and astrocytes can produce reactive oxygen species, leading to oxidative stress, which can damage neurons and contribute to cognitive decline [10]. In addition, glial activation can also inhibit neurogenesis in the hippocampus, further impacting cognitive function [11]. As a result, microglia-derived inflammatory factors can interfere with the process of long-term potentiation (LTP), a key mechanism for learning and memory formation, leading to cognitive deficits [12].

This study aims to explore these complex relationships, investigating how these molecular and cellular modifications within the brain contribute to the behavioral and cognitive manifestations observed in neuropathic pain. Understanding these mechanisms could facilitate the development of novel therapeutic strategies targeting the underlying cellular processes contributing to neuropathic-pain-related behavioral and cognitive deficits.

## 2. Results

### 2.1. Behavioral Performance in Mice with Neuropathic Pain

**Figure 1:**
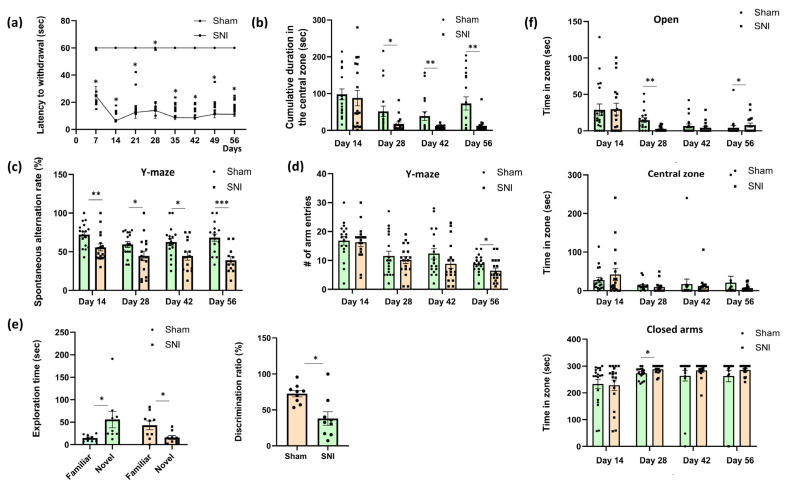
Behavioral performance in mice with neuropathic pain. (**a**) Cold allodynia: the moment of the first withdrawal of the injured paw from the cold (+4 °C) plate, sec. Mean ± SEM, n = 9 (number of animals per group), * *p* < 0.001 (compared to Sham). (**b**) The total amount of time spent in the center zone of the open field arena, sec. Mean ± SEM, n = 9 (number of animals per group). (**c**) Spontaneous alternation rate in the Y-maze, %. Mean ± SEM, n = 12–17 (number of test sessions per group; only test sessions with five or more arm entries were included in the analysis). (**d**) Locomotor activity in the Y-maze: number of arm entries. Mean ± SEM, n = 18 (number of test sessions per group). (**e**) The novel object recognition test. Left: exploration times for new and familiar objects for mice in “Sham” and “SNI” groups, sec. Right: recognition index, %. Mean ± SEM, n = 9 (number of animals per group). (**f**) Time spent in closed, open, and central zones of the elevated plus maze, sec. Mean ± SEM, n = 9 (number of animals per group). Student’s *t*-test, * *p* < 0.05, ** *p* < 0.01, *** *p* < 0.001.

Thermal allodynia, or the experience of pain when exposed to cold or thermal stimuli that ordinarily do not induce pain, is recognized as one of the symptoms of neuropathic pain [13]. We employed the “cold plate” method to assess thermal allodynia. Paw withdrawal latency in the cold plate test was significantly reduced in SNI animals compared to animals in a “Sham” control group, indicating the development of cold allodynia (Figure 1a). A marked decrease in latency was observed starting from day 7, with the most pronounced difference being observed on day 14, when SNI mice exhibited a significantly shorter response time relative to the control group (*p* < 0.001). This heightened sensitivity to cold persisted throughout the observation period, suggesting sustained neuropathic pain following nerve injury. There was no evidence of thermal allodynia in the “Sham” group.

Open field testing was used to measure anxiety, and the total amount of time spent in the center zone was computed. An increase in anxiety is correlated with a reduction in time spent in the center zone [14]. From day 28 to day 56 of observation, we observed a decline in this parameter in the “SNI” group relative to the “Sham” group. This implied that, even though anxiety remained at baseline levels during the first few weeks, prolonged exposure to pain brought on by nerve damage led to elevated anxiety (Figure 1b).

The Y-maze showed notable variations on day 14 (72.04 ± 3.89 “Sham” vs. 55.55 ± 4.66—“SNI”, *p* = 0.001), day 28 (59.30 ± 3.85—“Sham” vs. 44.59 ± 6.46—“SNI”, *p* = 0.048), day 42 (62.55 ± 5.06—“Sham” vs. 44.49 ± 5.66—“SNI”, *p* = 0.02) and day 56 (68.21 ± 4.72—“Sham” vs. 38.82 ± 4.95—“SNI”, *p* = 0.0002). Neuropathic pain induced by SNI significantly impairs spatial working memory, as evidenced by a sustained reduction in spontaneous alternation performance in the Y-maze on days 14, 28, 42, and 56 post-surgery compared to Sham controls. These findings indicate that SNI leads to persistent cognitive dysfunction that may progress over time (Figure 1c).

In addition, locomotor activity was determined in the Y-maze by counting the number of entrances to the arms of the maze (Figure 1d). On day 56 post-surgery, animals in the SNI group demonstrated a statistically significant, yet modest reduction in the total number of arm entries in the Y-maze, indicating a slight decline in locomotor activity at this late time point. This reduction may reflect subtle behavioral changes associated with the chronic pain state, such as decreased motivation or increased emotional distress. Importantly, the number of entries in SNI mice remained well above the minimum threshold required for inclusion in the spontaneous alternation analysis, suggesting that the observed impairments in cognitive performance cannot be attributed solely to reduced movement. This methodological control minimizes the likelihood that impaired performance in spatial memory tasks reflects motor dysfunction. Furthermore, previous studies have demonstrated that the spared nerve injury (SNI) model does not substantially impair gross locomotor abilities, and that spontaneous alternation performance is a reliable measure of hippocampal-dependent cognition, relatively unaffected by minor variations in movement [15,16,17,18].

The Novel Object Recognition Test was then used to investigate long-term memory, another category of hippocampal-dependent processes. Eight weeks following surgery, the test was given once. The “SNI” group’s recognition index decreased, according to the results (64.80 ± 8.34—“Sham” vs. 33.26 ± 10.91—“SNI”, *p* = 0.02). The “SNI” group spent 42.22 ± 11.49 and 15.70 ± 5.98 s (*p* = 0.041) with the familiar and new objects, respectively, whereas the “Sham” group spent 14.71 ± 2.57 and 55.85 ± 19.51 (*p* = 0.047) (Figure 1e).

The elevated plus maze, which is commonly used to measure anxiety, yielded some intriguing findings as well. In pain-induced animals the time spent in the open arms decreased on day 28 (13.69 ± 3.75—“Sham” vs. 2.55 ± 0.93—“SNI”, *p* = 0.005), whereas the time spent in the closed arms increased (273.21 ± 5.63—“Sham” vs. 288.00 ± 4.17—“SNI”, *p* = 0.03), which is indicative of a rise in anxiety. The mice in both groups favored the closed arms over the open ones, as the findings demonstrate, but the mice with a nerve injury had a noticeably higher propensity to be in the closed arms. It is also intriguing that there were no notable differences across the groups at day 42. On day 56 post-surgery, SNI animals demonstrated a modest increase in open-arm exploration. Although this difference was not large, it was statistically significant (*p* = 0.023), suggesting a potential reduction in anxiety-like behavior during the late stage of neuropathic pain. This finding may reflect behavioral adaptation or altered emotional processing over time. However, it should be interpreted with caution, as chronic pain is typically associated with increased anxiety, and the observed shift might also have resulted from non-specific factors such as habituation to the testing environment or changes in risk assessment behavior (Figure 1f).

### 2.2. Effects of Neuropathic Pain on the Condition of Hippocampal Glial Cells

We utilized the microglial marker Iba-1 and the astroglial marker glial fibrillary acidic protein (GFAP) to evaluate the status of microglia and astroglia in the hippocampus in neuropathic pain [19,20]. Numbers of immunopositive cells in the ventral and dorsal regions of the hippocampus, as well as in the ipsilateral and contralateral regions, were counted independently. In this instance, the ipsilateral and contralateral hippocampal regions showed notable differences, while the ventral and dorsal hippocampal regions showed unidirectional changes. As a result, we thought it would be feasible to integrate the data from the ventral and dorsal hippocampal regions.

**Figure 2:**
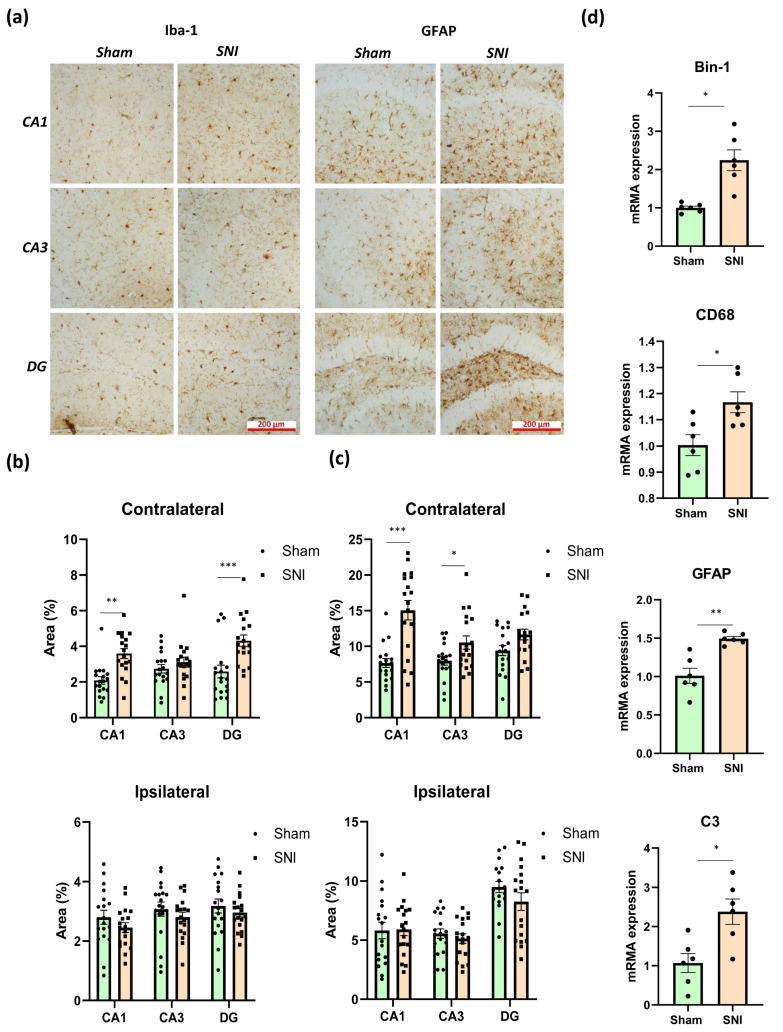
Effects of neuropathic pain on the condition of hippocampal glial cells. (**a**) Representative images of immunopositively stained microglia and astrocytes in contralateral hippocampi, scale bar −200 μm. (**b**) Iba-1-immunopositive areas within the contra- and ipsilateral hippocampi, %. Mean ± SEM, n = 18 (number of analyzed images per group, three slices from a single animal). (**c**) GFAP-immunopositive areas within the contra- and ipsilateral hippocampi, %. Mean ± SEM, n = 18 (number of analyzed slices per group, three slices from a single animal). (**d**) Real-time PCR-measured quantitative content of hippocampal microglial markers, relative mRNA levels. Mean ± SEM, n = 6 (number of analyzed samples per group, two samples from a single animal). Student’s *t*-test, * *p* < 0.05, ** *p* < 0.01, *** *p* < 0.001.

The contralateral hippocampus displayed more noticeable alterations, although the investigation was conducted in both the ipsilateral and contralateral hippocampus. Representative images of immunopositively stained microglia and astrocytes are displayed in Figure 2a. In neuropathic pain, the contralateral hippocampus exhibited an accumulation of the microglia marker Iba-1, according to immunohistochemical detection, whereas the ipsilateral hippocampus revealed no discernible alterations. In the CA1 region, the percentage of immunopositive microglia was 2.09 ± 0.24—“Sham” compared to 3.57 ± 0.14—“SNI” (*p* = 0.0006); in the DG region, the percentage was 2.06 ± 0.20—“Sham” compared to 4.28 ± 0.37—“SNI” (*p* < 0.0001) (Figure 2b). It is well established that the microglial protein BIN1 is crucial in controlling how the brain reacts to inflammatory processes and microglial phenotypic alterations. We used PCR to examine the amount of BIN1 in the hippocampus and discovered that peripheral neurotrauma increased its content: 1.01 ± 0.05—“Sham” vs. 2.24 ± 0.33—“SNI” (*p* = 0.01) (Figure 2d). CD68, a protein highly expressed in microglia, plays a crucial role in the activation of these immune cells within the hippocampus during neuropathic pain. In this context, we used the PCR technique to examine the amount of CD68 in the contralateral hippocampus. As a result, we found that SNI slightly upregulated CD68 within the hippocampus: 1.00 ± 0.04—“Sham” vs. 1.16 ± 0.04—“SNI” (*p* = 0.02) (Figure 2d).

Immunohistochemical detection of GFAP, which is used to examine astroglial activity in the hippocampus, revealed that GFAP accumulated mostly in CA1 and CA3 in the contralateral hippocampus. The proportion of immunopositive astrocytes in the CA1 region was 7.66 ± 0.48—“Sham” vs. 15.06 ± 1.07—“SNI” (*p* = 0.0001), while in the CA3 region it was 7.97 ± 0.47—“Sham” vs. 10.54 ± 0.73—“SNI” (*p* = 0.04) (Figure 2c). GFAP was also found to be upregulated in the hippocampus by PCR, with a result of 1.01 ± 0.12—“Sham” vs. 1.49 ± 0.03—“SNI” (*p* = 0.01) being obtained (Figure 2d).

The pathogenesis of neuropathic pain is known to involve the astrocytic marker complement 3 (C3). Astrocytes are the primary source of C3 expression, while microglia and neurons express their receptors. By releasing cytokines, including TNF, C1q, and Il-1α, microglia have been shown to stimulate astrocytes to produce C3. Astroglia then control microglial activity through the C3/C3aR pathway. Levels of proinflammatory microglia M1 decrease while those of anti-inflammatory microglia M2 increase when the C3-C3aR pathway is blocked, according to the underlying molecular mechanism [21]. Our research showed that neuropathic pain increased C3 levels within the hippocampus: 1.06 ± 0.29—“Sham” vs. 2.38 ± 0.39—“SNI” (*p* = 0.031) (Figure 2d).

### 2.3. Hippocampus Neurogenesis in Neuropathic Pain

**Figure 3:**
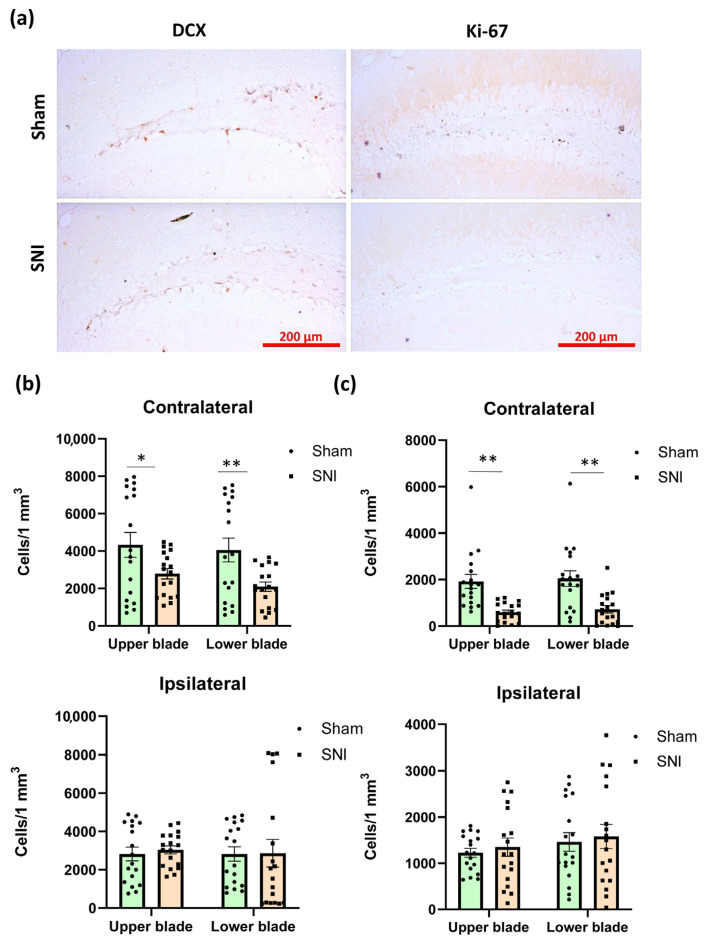
Hippocampal neurogenesis in neuropathic pain. (**a**) Representative images of doublecortin (DCX)- and Ki67-positive neurons in the DG SGZ in contralateral hippocampi. (**b**) Plot showing the number of DCX-positive neurons in DG SGZ. UB—upper blade, LB—lower blade. Mean ± SEM, n = 18 (number of analyzed images per group, three slices from a single animal). (**c**) Plot showing the number of Ki67-positive neurons in the DG SGZ. Mean ± SEM, n = 18 (number of analyzed images per group, three slices from a single animal). Mean ± SEM. Student’s *t*-test, * *p* < 0.05, ** *p* < 0.01.

Proliferation markers and markers of newly formed neurons are both used to assess hippocampal neurogenesis [22] (Figure 3a). To evaluate neurogenic activity in the hippocampus, we measured the density of doublecortin-positive (DCX) cells in the subgranular zone of the dentate gyrus (DG SGZ). As expected, we observed a decrease in newly formed neurons, but only in the contralateral hippocampus.

Obtained values were 4330.86 ± 723.82 for the “Sham” group and 2784.91 ± 292.01 for the “SNI” group (*p* = 0.03) within the upper blade and 4053.76 ± 422.03 for the “Sham” group and 2097.465 ± 689.77 for the “SNI” group (*p* = 0.007) within the lower blade (Figure 3b). We also looked at how many cells in the DG SGZ were Ki-67-positive (proliferation marker). Immunopositive cells in the DG SGZ are most likely proliferating neurons, as Ki-67 is a sign of proliferation. In the DG SGZ, there were 245.89 ± 50.20 Ki-67-positive cells in the “Sham” group and 98.05 ± 22.88 in the “SNI” group (*p* = 0.008) (Figure 3c).

### 2.4. The Impact of Neuropathic Pain on Levels of Synaptic Plasticity-Related Proteins

Numerous neurological dysfunctions have been linked to postsynaptic density protein 95 (PSD-95) [23,24]. PSD-95 in the spinal cord has been implicated in the pathophysiology of neuropathic pain [24], while PSD-95 in the cortex has been shown to be crucial for learning, memory, and neuroplasticity [23]. This protein also helps NMDA and AMPA glutamate receptors attach to signaling proteins and the neuronal cytoskeleton. During synaptic plasticity, PSD-95 controls glutamate receptor endocytosis and surface expression [24].

**Figure 4:**
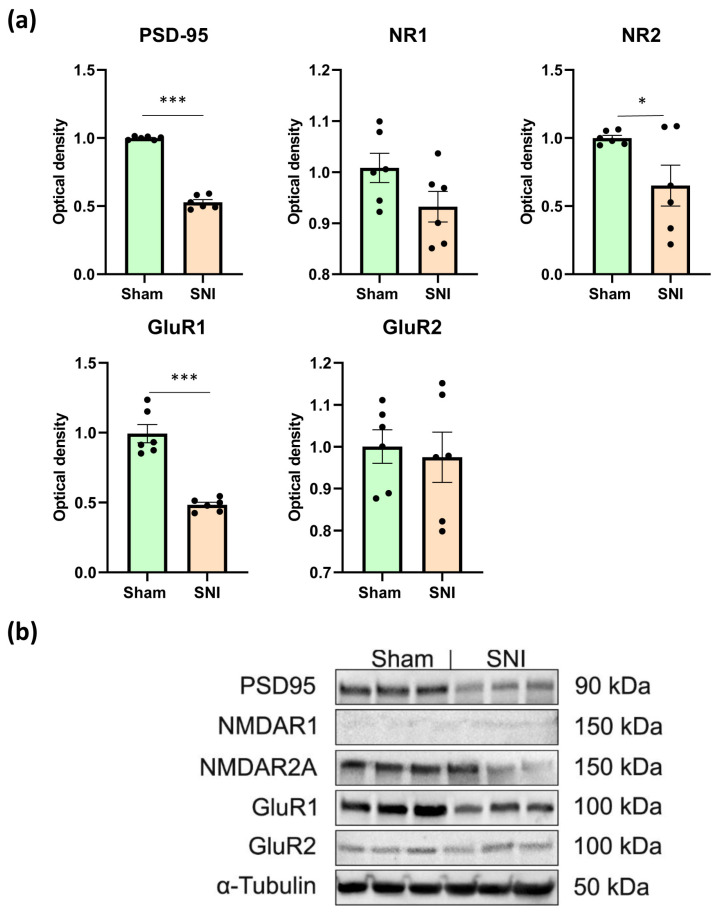
Western blot analysis of synaptic plasticity-related protein levels. (**a**) Relative quantities of PSD95, NMDAR1, NMDAR2A, GluR1, and GluR2 in hippocampal lysates determined with Western blot, mean ± SEM, n = 6 (number of analyzed samples per group, two samples from a single animal). Student’s *t*-test, * *p* < 0.05, *** *p* < 0.001. (**b**) Levels of PSD95 (90 kDa), NMDAR1 (150 kDa), NMDAR2A (150 kDa), GluR1 (100 kDa), and GluR2 (100 kDa) were determined in hippocampal lysates, with β-tubulin protein expression used as a loading control. The full, uncropped blot images are provided in Supplementary Figures S1–S3.

We used Western blotting to measure amounts of PSD-95, NMDA, and AMPA receptors in the hippocampus, in order to comprehend the type of behavioral abnormalities associated with neuropathic pain. Western blotting was conducted on contralateral hippocampal lysates, as these regions showed the most significant alterations in glial activity and neurogenesis. Consequently, we discovered that neuropathic pain lowered PSD-95 concentration by around two times (0.99 ± 0.005—“Sham” vs. 0.52 ± 0.022—“SNI”, *p* < 0.0001). The NR2 subunits of NMDA receptors and the GluR1 subunits of AMPA receptors also showed a consistent decline (0.99 ± 0.02—“Sham” vs. 0.65 ± 0.035—“SNI”, *p* = 0.03 and 1.00 ± 0.08—“Sham” vs. 0.48 ± 0.02—“SNI”, *p* < 0.0001). However, pain did not affect the levels of NR1 or GluR2 (Figure 4a). Representative Western blot images are presented in Figure 4b.

## 3. Discussion

This study expands the understanding of hippocampal involvement in neuropathic pain by providing a temporally resolved, multimodal analysis of behavioral and molecular alterations following spared nerve injury (SNI). In line with translational standards, chronic pain in rodent models is typically defined as pain persisting for longer than three weeks post-injury [25,26], which reflects the chronicity threshold adapted to rodent lifespan and neurobiology. Our findings, including sustained allodynia and hippocampal alterations beyond week 4, are consistent with this definition and support the classification of this model as chronic neuropathic pain.

Although the initial pathology in neuropathic pain arises from peripheral nerve injury, our results clearly demonstrate that sustained nociceptive signaling induces profound and progressive alterations in supraspinal brain structures, particularly in the hippocampus. While cognitive and affective disturbances have been reported in rodent models of neuropathic pain, our study is, to our knowledge, the first to combine spatial memory, object recognition, and anxiety-related assessments across multiple time points after SNI. This longitudinal behavioral profile, matched with hippocampal molecular and cellular analysis within the same animals, reveals a coordinated trajectory from peripheral injury to cognitive-affective dysfunction. We demonstrate that structural and molecular alterations, such as glial activation and impaired neurogenesis, are lateralized to the hippocampus contralateral to nerve injury. In previous studies involving SNI models, spatial specificity has rarely been addressed. Our study is the first to report upregulation of BIN1 in the hippocampus in the context of neuropathic pain, which may represent a novel marker of microglial activation and synaptic remodeling, linking neuroinflammation with cognitive dysfunction. In addition to general microglial burden (Iba1), we assessed CD68 expression as a marker of phagocytic activation. While CD68 has been widely used in models of neurodegeneration, its application in the hippocampus under neuropathic pain conditions remains scarce. Our findings reveal a selective increase in CD68-positive microglia in the contralateral hippocampus, supporting an active role of microglia in synaptic remodeling. When combined with BIN1 upregulation, this suggests a coordinated microglial response involving both inflammatory and structural components. We further report a novel increase in astrocytic C3 expression within the contralateral hippocampus following SNI, marking the emergence of neurotoxic A1 astrocyte activation [27]. Although enhanced C3/C3aR signaling has been implicated in spinal glial–immune crosstalk in neuropathic pain models [28], its elevation in hippocampal astrocytes after peripheral nerve injury has not been previously demonstrated. Finally, by integrating behavioral, immunohistochemical, and biochemical analyses within a longitudinal design, our work provides a coherent view of how peripheral neuropathy gradually reshapes supraspinal circuits involved in cognition and affect.

In addition to classical sensory symptoms such as spontaneous pain, hyperalgesia, and allodynia, chronic neuropathic pain is often accompanied by complex neuropsychiatric manifestations, including heightened anxiety, depressive-like behaviors, impaired cognitive functions, and disrupted sleep patterns [29]. It is a fact that the brain’s supraspinal areas play a part in the processing and transmission of pain signals [30]. More than 50 years ago, [31] Melzack and Casey proposed that pain signals are processed by the brain’s neuromatrix, a huge and hierarchically connected network of neurons. According to this view, pain is a complex experience that has elements connected to affective-motivational, cognitive-evaluative, and sensory-discriminative components. The affective and motivational aspects of pain encourage actions necessary to repel the threat and bring about healing, whereas the sensory-discriminatory aspect of pain warns the body of danger. The cognitive-evaluative aspect of pain involves a critical evaluation and analysis of pain experiences to prevent such situations in the future, and the perception of future pain sensations is also influenced by past pain experiences. The hippocampus, a component of the limbic system, is involved in memory, learning, and sensory integration, and its role in processing pain has become better understood in recent years [32,33]. This region is involved in the affective-motivational aspect of pain perception, as evidenced in numerous published works, including behavioral, biochemical, and electrophysiological studies [32]. Our research validates classification of the hippocampus as a supraspinal pain center. In addition, the hippocampus, a region of the brain that is highly susceptible to a variety of negative external influences, experiences maladaptive changes in neuropathic pain, impairing both higher neurological and sensory systems. In this study, impairment of hippocampus-dependent functions became manifest two to five weeks after the procedure. During this time period, we observed affective abnormalities, manifested as an increase in anxiety; and cognitive impairment, manifested as a decline in working spatial and long-term memory. Thermal allodynia, on the other hand, started to manifest immediately, peaking at week two of the study and continuing until week eight.

A decrease in the amount of time spent in the open field’s center at week four of testing may show that the ventral hippocampus was processing the pain signal. In addition, and in line with the open field results, the assessment of anxiety in the elevated plus maze at week four also showed a decrease in the amount of time spent in the open arms. However, by the eighth week of testing, the animals started to favor open arms, which could be a sign that impulsivity or risk-taking behaviors were developing. Although such behavior in TBI has been described many times, we have not found any references in the literature to such behavior in mice with induced neuropathic pain [34].

The observed changes in working spatial memory and long-term memory may indicate that the dorsal hippocampus plays a role in the cognitive assessment of pain experience and that there are projections to the dorsal hippocampus that transmit information about the pain signal [35]. Impaired working spatial memory was noted two weeks after surgery and this continued until the end of the experiment; moreover, the “Novel Objects Recognition” test, which measured long-term memory at week eight of the experiment, revealed a significant decrease in the indicator. Therefore, because neuropathic pain requires significant neuropathic changes, it is clear that the development of cognitive changes is delayed upon induction of neuropathic pain.

In our study, impairments in spatial working and long-term memory were accompanied by a decrease in hippocampal neurogenesis. Neurogenesis in the hippocampal dentate gyrus is highly sensitive to peripheral nerve injury, and its suppression is considered a key mechanism linking chronic pain to cognitive and affective impairments. Several factors modulate this response. Proinflammatory cytokines (e.g., TNF-α, IL-1β) released by activated microglia inhibit neural progenitor proliferation and differentiation via the NF-κB and JNK signaling pathways [36]. In addition, glutamatergic dysfunction, including reduced NMDA receptor signaling, alters calcium dynamics essential for neurogenic niche maintenance [37]. Furthermore, hypothalamic–pituitary–adrenal (HPA) axis dysregulation in chronic pain elevates glucocorticoids, which are known suppressors of neurogenesis [38]. Notably, these mechanisms appear time-dependent and can persist beyond the resolution of peripheral hypersensitivity, suggesting that long-term hippocampal plasticity is not merely a downstream effect but a sustained consequence of neuroimmune dysregulation.

According to numerous studies, hippocampal neurogenesis may be important for working memory and long-term memory tasks that involve the discrimination of similar cues or that involve long temporal delays [39]. However, the relationship between working memory and hippocampal neurogenesis is not straightforward. Some studies have even shown paradoxical results. For example, the authors of [40] found that suppressing neurogenesis can sometimes improve certain aspects of working memory. Nevertheless, the ability to distinguish between similar experiences is likely to be important for some forms of working memory, particularly those that require the discrimination of similar cues [41]. The ability to distinguish between the three arms of the Y-maze, especially if subtle changes are introduced to the environment, relies on the brain’s ability to differentiate spatial information. Therefore, to some degree, the Y-maze does require the animal to use pattern separation. In addition, hippocampal neurogenesis plays a dynamic role in long-term memory in rodents, contributing both to the formation of detailed memories and to the ongoing modulation of existing memory representations. In rats, for instance, the suppression of adult neurogenesis has been shown to inhibit the enhancement of long-term recognition memory through contextual enrichment [42]. It has also been shown that the hippocampus influences the performance of tasks related to long-term recognition memory [43], and that hippocampal neurogenesis is linked with long-term recognition memory, particularly when contextual information and pattern separation are involved [44]. However, the relationship between impaired hippocampal-dependent memory tasks and hippocampal neurogenesis in rodents is an active area of further research. As previously noted, the impairment of neurogenesis may be driven by a neuroinflammatory response, with microglia releasing a broad range of proinflammatory factors. Because neuroinflammation is recognized as an etiological factor and/or a pathogenetic link in the majority of neurological and neurodegenerative disorders [45], we were first interested in this process. Numerous studies on neuropathic pain have indicated a substantial neuroinflammatory response in the supraspinal regions [46] as well as at the spinal cord level [47]. In this study, we observed an increase in the production of neuroinflammation markers in the hippocampus, mainly in the contralateral region. Anatomical characteristics of the ascending nociceptive pathways’ placement may be linked to this lateralization. The pain signal is transmitted from the spinal cord to the brainstem and beyond via several ascending pathways, including the spinothalamic tract and the spinoreticular tract [48]. The secondary neurons in the dorsal horn cross to the contralateral side of the spinal cord at the anterior white commissure and ascend through the spinothalamic tract. These neurons project to the thalamus, a relay center that directs sensory information to the appropriate cortical areas. The thalamus acts as a relay station, processing pain signals and transmitting them to various brain regions, including the somatosensory cortex, which processes the sensory aspect of pain, and the limbic system, which processes the emotional component. The thalamus also sends projections to the prefrontal cortex (involved in cognitive control) and the hypothalamus (involved in autonomic response to pain). Signals from the thalamus are relayed to the cingulate cortex and other areas of the limbic system, which then influence hippocampal function [49].

Microglia and astroglia, two types of glial cells in the central nervous system (CNS), play a significant role in pain processing within the hippocampus. Specifically, proinflammatory cytokines released by M1 microglia (e.g., TNF-α, IL-1β) and reactive astrocytes have been shown to modulate synaptic receptor trafficking and suppress glutamatergic transmission through receptor internalization or degradation [46,50]. Such glia-mediated dampening of excitatory synaptic signaling may contribute to altered pain processing and emotional-affective dysregulation associated with neuropathic pain states, as the hippocampus is increasingly recognized as a key region in the modulation of pain, affect, and memory of pain.

It is important to note that glial responses to peripheral nerve injury are influenced by sex, strain, and the type of neuropathic model. For instance, male rodents often exhibit microglia-dependent mechanisms of pain hypersensitivity, whereas astrocytic or T-cell involvement is more prominent in females [51,52]. Mouse strain significantly impacts the changes observed in hippocampal glia during neuropathic pain due to strain-specific basal glia levels [53], genetic predisposition to neuropathic pain [54], differential immune responses [55], and variable glia-neuron interactions [56]. The choice of the spared nerve injury model also contributes to the observed pattern and duration of glial reactivity in the hippocampus. These variables should be considered when interpreting of our findings.

We found an increase in production of the microglial marker Iba-1 and of the proinflammatory microglia marker CD68. Previously, increases in microglial markers in SNI have been recorded in the prefrontal cortex [57,58] and in limbic system regions [59,60]; however, there are currently very few studies devoted to microglia changes in the hippocampus in neuropathic pain. Interestingly, we found an increase in Bin-1 in the hippocampus in neuropathic pain. BIN1 is a crucial modulator of the activation of proinflammatory and disease-related responses in microglia expressed in both neurons and glial cells [60]. Neuronal BIN1 is located in the presynaptic terminals of the mouse brain. It plays an important role in excitatory neurotransmission by controlling the dynamics of synaptic vesicles. Deficiency in neuronal BIN1 causes selective impairment of spatial learning and memory [61]. In microglial cells, BIN1 regulates key pathways and functions under normal and pathological conditions [60]. The increased BIN1 immunoreactivity found in our study may serve as a marker of microglial activation in progressive pathology. Additionally, Bin1 is involved in protein complexes with GluR1 [62], regulating AMPA receptor surface expression and trafficking, as well as AMPA receptor-mediated synaptic transmission [63]. This is consistent with our data demonstrating decreased GluR1 expression in the hippocampus following pain induction. Additionally, the increase in Bin1 may be associated with the decreased NR2 receptor expression that we also observed in the hippocampus, although this requires further investigation. Interestingly, the decrease in NR1 was not significant, while the decrease in the less abundant NMDA receptor subunit NR2 was significant. This is likely due to the higher prevalence of the NR2 subunit of NMDA receptors in the limbic system and, particularly, within the hippocampus [64]. Such a pronounced decrease in NMDAR2 is essential for the impairment of hippocampus-dependent cognitive functions. It is known that NMDAR2B plays an important role in the induction of LTP, which is extremely important for learning and memory [65]. Thus, decreases in the concentrations of NMDA and AMPA receptors in the hippocampus may underlie the memory impairment which we observed in mice with neuropathic pain syndrome. Previous studies have already investigated changes in NMDA receptors in the brain as a result of neuropathic pain, but ours is the first to pay direct attention to the hippocampus. For example, an increase in NR2B expression was shown in SNI in rats in the central nucleus of the amygdala [65], which plays a key role in the regulation of emotional processes. The observed decrease in glutamate receptor expression in the hippocampus is apparently the result of strong reciprocal connectivity between the amygdala and the hippocampal formation [66].

PSD95 is another crucial protein that maintains memory function and cognitive processes. PSD-95 has consistently been shown to play an important role in the development, function, and plasticity of excitatory synapses through direct or indirect interactions with glutamate receptors NMDAR and AMPAR, other scaffolding, and signaling proteins [67]. However, only a few studies have reported PSD-95 expression in the central nervous system in neuropathic pain. Neuropathic pain, for instance, has been linked to increased PSD95 levels in the anterior cingulate cortex (ACC) and spinal cord [68]. This enhances the interaction between NMDA receptors (primarily the NR2B subunit) and the postsynaptic scaffolding. The formation and maintenance of central sensitization, a major mechanism underlying chronic pain states triggered by nerve injury, are both facilitated by this phenomenon. In essence, elevated PSD-95 expression increases neuronal excitability, which in turn amplifies pain signals. However, as our research demonstrates, neuropathic pain mostly results in a reduction in PSD-95 expression and glutamate receptor levels in the hippocampus. As demonstrated in the LPS-induced neuroinflammation paradigm, neuroinflammation is most likely the cause of the decrease in PSD-95 [67]. These changes in the expression and function of PSD-95, NMDA receptors, and AMPA receptors are thought to contribute to the development of memory deficit and anxiety by decreasing the excitability of hippocampal neurons and by reducing the transmission of pain signals to the brain.

In summary, our data underscore the significant roles played by hippocampal neuroinflammation, disrupted glutamatergic signaling, and impaired adult neurogenesis in mediating the cognitive and affective sequelae of chronic neuropathic pain. Peripheral nerve injury initiates a cascade of cellular and molecular events within the hippocampus that likely underlie observed behavioral and cognitive disturbances. One major pathway involves injury-induced M1 microglia activation followed by proinflammatory cytokines release [69]. Proinflammatory factors are known to disrupt synaptic plasticity by downregulating PSD-95 and AMPA/NMDA receptor subunits, thereby impairing long-term potentiation (LTP) and memory function [70]. Additionally, astrocytes, marked by GFAP, modulate extracellular glutamate and inflammatory mediators, affecting neuronal excitability [71]. Complement signaling components such as C1q and C3, classically involved in developmental synaptic pruning, are re-expressed during chronic pain states and may drive aberrant microglia-mediated elimination of functional synapses [72]. In this context, our novel finding of BIN1 upregulation in the hippocampus is noteworthy. BIN1, a membrane-sculpting protein implicated in neurodegenerative disorders, has been shown to regulate microglial priming and synaptic integrity [60,61], suggesting that it may serve as a mechanistic link between glial activation and synaptic remodeling in neuropathic pain. These results suggest that hippocampal glial activation and loss of synaptic proteins jointly mediate the cognitive and affective symptoms seen after SNI. Microglia- and astrocyte-derived inflammatory signals may disrupt glutamatergic transmission and neuroplasticity, linking neuroinflammation to behavioral dysfunction.

Although our study was conducted using the SNI model, the observed hippocampal alterations, namely, glial activation, synaptic protein downregulation, and impaired neurogenesis, are not unique to this model. Similar changes have been reported in other well-established models of neuropathic pain, including chronic constriction injury (CCI), partial sciatic nerve ligation (PSNL), and spinal nerve ligation (SNL). For example, CCI leads to impairments in spatial and recognition memory that correlate with decreased hippocampal PSD-95 and GluR1 expression, indicating synaptic dysfunction [73,74]. PSNL has also been shown to induce prominent glial activation in the hippocampus, with associated cognitive deficits [75]. These findings support the interpretation that the hippocampal disturbances reported here are representative of a shared supraspinal maladaptive response to chronic peripheral nerve injury, rather than an SNI-specific phenomenon.

These findings extend the traditional view of pain processing by incorporating supraspinal dysfunction into the pathophysiological model. Importantly, they highlight potential targets for pharmacological and therapeutic interventions aimed at mitigating not only the nociceptive but also the emotional and cognitive burdens of chronic pain.

One limitation of our study is the exclusive use of male mice. Given known sex differences in pain processing and hippocampal responses to stress and inflammation, future studies should include both sexes to enhance the translational relevance and generalizability of findings.

## 4. Materials and Methods

### 4.1. Animals

In the study, 18 male C57Bl/6 mice aged three months were raised in the vivarium of the Russian Academy of Sciences’ Far Eastern Branch’s National Scientific Center for Marine Biology in Vladivostok, Russia. There were three or four mice in each cage. We employed male C57BL/6J mice, a well-established inbred strain characterized by stable and reproducible development of mechanical and thermal hypersensitivity after nerve injury (CCI/SNI) without substrain variability. The genetic uniformity of this strain enhances reproducibility, and its widespread use enables future genetic and transgenic extensions. Additionally, C57BL/6J mice exhibit robust thermal nociceptive responses, making them reliable for neuropathic pain paradigms [76]. Mice were housed on a 12 h light/dark cycle and had unrestricted access to both food and water. The vivarium’s air temperature was 23 ± 2 °C, and its humidity was 55 ± 15%. According to the Guidelines for the Welfare of Laboratory Animals and Directive of the Council of the European Community 2010/63/EU, the Animal Ethics Committee of the National Scientific Center for Marine Biology, Far East Branch of the Russian Academy of Sciences (No. 1/2024, 15 January 2024) has approved all animal manipulations and surgeries.

### 4.2. Surgery

Neuropathic pain was induced using the spared nerve injury model (SNI) [77]. The mice were sedated with isoflurane using a rodent anesthetic vaporizer (VetFloTM, Kent Scientific Corporation, Torrington, CT, USA). Following the animal’s anesthesia, the right sciatic nerve was exposed, and the tibial and common peroneal nerves, two of the sciatic nerve’s three terminal branches, were securely tied with a 4-0 silk suture (Ethicon, Irvine, CA, USA). The ligatures were tightened until the limb started to tremble slightly. The ligated branches were transected distal to the ligature, and 2 mm of each distal nerve stump was removed. In the “Sham” group, the sciatic nerve and its branches were exposed but not transected or ligated. Each animal’s skin and muscles were sutured separately using a 4–0 silk suture (Ethicon, Irvine, CA, USA).

### 4.3. Behavioral Tests

All behavioral tests were conducted between 7:00 and 19:00, during the light portion of the day/night cycle. After each animal was tested, the test equipment was meticulously cleaned with 10% ethanol to lessen smell cues. To avoid stress from the unfamiliar surroundings, mice were kept in the test apparatus for ten minutes every day for three days before the test day. On the day of the test, the mice spent two hours in the test room in their home cages. Every week, thermal allodynia was evaluated. Memory tests were conducted in the 56 days after the operation.

#### 4.3.1. Thermal Allodynia

Thermal allodynia was measured using a cold/hot plate analgesiometer (Columbus Instruments, Columbus, OH, USA). A 30 × 30 cm metal plate was used for the studies, which were conducted in a room with acrylic walls that were 30 cm high [78]. The testing time was 60 s, and the temperature of the cold plate was +4 °C. The time elapsed before the mouse first removed its wounded hind paw from the plate after being placed on it was noted. This test was conducted weekly.

#### 4.3.2. Y-Maze Testing

The working memory of mice was evaluated using the Y-maze spontaneous alternation test [79]. For this experiment we used a Y-shaped acrylic glass maze (30 × 10 × 20 cm) with three identical arms. The mouse was put in the center of the maze and allowed five minutes to explore the area freely. The sequence of the entrances was noted, in order to calculate the spontaneous alternation rate. It was considered that the entrance had been made when all four of the animal’s paws were within the arm. This test was conducted four times every two weeks before the animals were sacrificed. As a criterion for data inclusion, we excluded Y-maze sessions in which mice made fewer than five arm entries, thereby minimizing the influence of reduced locomotor activity on spontaneous alternation performance. The following formula was utilized to determine the spontaneous alternation rate:

SAR = N/A ∗ 100%(1)

where SAR is the spontaneous alternation rate, N is the quantity of consecutive entries into the three nonrepeating arms, and A is the total number of possible alternations.

#### 4.3.3. Open Field Test

Open field testing [80] was used to measure anxiety-like behavior. For five minutes, each animal was positioned in the middle of a circular plexiglass arena that measured 50 cm in circumference and 40 cm in height. Each of the six sectors that made up the arena featured three parts (one central, and two peripheral). The center and peripheral zones delineated the arena space. A video camera positioned above the apparatus was used to capture the animal’s activity, and the quantity of crossed squares was tallied.

#### 4.3.4. Novel Object Recognition Test

The novel object recognition (NOR) test was conducted using Bevins and Beshear’s methodology [81]. The animals were pre-trained, or familiarized and habituated, in a test apparatus the day before the test. Each mouse was positioned in the middle of the arena, with two identical objects on either side, and given ten minutes to explore during the first stage (training session). After that, the animal spent twenty-four hours in its home cage. Long-term memory was assessed using this retention interval. In the second stage, the mouse was once again positioned in the middle of the arena for five minutes; then, one of the items was swapped out for a new one. A recording device placed above the object under analysis was used to capture mouse behavior on video. The core criteria for object exploration were concerned with active investigation, as follows: the mouse must (1) direct its nose toward the object within 1–2 cm; (2) sniff, touch, or manipulate the object. We determined the discrimination index by dividing the amount of time spent examining a new object by the overall amount of time spent exploring the objects. After each animal was tested, 70% ethanol was used to properly clean the items and testing apparatus.

#### 4.3.5. Elevated Plus Maze Test

An elevated plus maze (Panlab/Harvard Apparatus, Holliston, MA, USA) with two closed arms (15 cm in height, 30 cm in length, and 5 cm in width) and two open arms (1 cm in height, 30 cm in length, and 5 cm in width) was utilized in the experiment [82]. The mouse was left to explore the area freely and investigate the arms after being positioned on the center plate. Mouse behavior was tracked using a video camera equipped with a video tracking system and SMART 3.0 software (Panlab/Harvard Apparatus, USA). The number of entries to the maze’s zone, and the times spent by the mouse in the center, open arms, and closed arms of the maze were all monitored and documented. The behavioral abnormalities revealed in this test can be interpreted as modifications in the neural defense systems that make the animal either more avoidant or more risk-averse. A risk-taking behavior is indicated by an increase in the quantity of time spent in the open arms of the maze.

### 4.4. Immunohistochemical Studies

Animals were withdrawn from the experiment 8 weeks after the surgery, immediately after behavioral tests were performed. Using a rodent anesthesia vaporizer (VetFloTM, Kent Scientific Corporation, Torrington, CT, USA) with a mouse mask, the animals were deeply sedated with isoflurane (Laboratories Kari-zoo, S.A., Barcelona, Spain) in order to extract their brains and perform immunohistochemistry. PBS (~4 °C), pH 7.2, was transcardially infused into anesthetized animals. Following fixation, the brain was embedded in paraffin blocks and cleaned with PBS (pH 7.2). Sagittal slices 10 µm thick were created using a Leica RM 2245 microtome (Leica, Wetzlar, Germany). The slides were treated in a 0.3% hydrogen peroxide solution for five minutes, in order to inhibit endogenous peroxidase activity. By incubating with 5% BSA in PBS for one hour, non-specific antibody binding was blocked. A solution of primary and secondary antibodies conjugated with horseradish peroxidase—anti-rabbit, 1:200, PI-1000-1; anti-mouse, 1:200, PI-2000-1—from Vector Laboratories, San Francisco, CA, USA, was used for further incubation. ImmPACTTM DAB peroxidase chromogen substrate (SK-4105, Vector Laboratories, San Francisco, CA, USA) was used for staining. Following a 0.1 M PBS (pH 7.2) wash, the slides were dehydrated and placed in VectaMount Permanent Mounting Medium (H-5000, Vector Laboratories). Rabbit polyclonal antibodies to Iba-1 (1:500, ab108539, Abcam, Cambridge, UK), rabbit polyclonal antibodies to GFAP (1:1000, ab7260; Abcam, Cambridge, UK), rabbit polyclonal antibodies to Ki67 (1:1000, ab15580; Abcam, Cambridge, UK), and rabbit polyclonal antibodies to doublecortin (1:1000, ab18723, Abcam, Cambridge, UK) were utilized. Slides were seen using a Zeiss Axio Imager microscope using an AxioCam 503 color camera and Axio-Vision software Rel. 4.9 (Carl Zeiss, Oberkochen, Germany). ImageJ software 1.53t (NIH, Bethesda, MD, USA) was used for image processing and analysis. The background was removed from each micrograph after it was converted to an 8-bit image (rolling ball radius = 50), then thresholded. The percentage area was measured within manually drawn CA1, CA3, and DG ROIs. Every sixth slice was used to measure the immunopositive staining area and the density of immunopositive cells in the hippocampal CA1, CA3, and DG regions. The measurements and evaluation were performed by the researcher, who was unaware of the identities of the images.

### 4.5. Real-Time PCR

Total RNA from the hippocampus was isolated and purified using ExtractRNA kits (Evrogen, Moscow, Russia) and CleanRNA Standard kits (Evrogen, Moscow, Russia). The resulting RNA was analyzed by agarose gel electrophoresis, and a spectrophotometer (Thermofisher, Waltham, MA, USA) was used to estimate the concentration. To synthesize the first strand of cDNA, 1 μg of total RNA and the ProtoScript^®^ II kit (New England Biolabs, UK) were used.

Real-time PCR (RT-PCR) was performed using the SYBR Green I RT-PCR kit (Evrogen, Moscow, Russia) and a CFX96 Real-Time PCR System (Bio-Rad, Hercules, CA, USA) with the following thermocycling parameters: 95 °C for 10 min (one cycle), then 95 °C for 15 s, 67 °C for 40 s, and 72 °C for 10 s (40 cycles), followed by a denaturation step to verify the amplification of a single product. The reaction was carried out in 20 μL buffer with 1 μL of cDNA, and the primer concentration was 0.5 μM. The same mixture without cDNA was used as a control for each pair of primers. We used beta-actin (Actb) and glyceraldehyde-3-phosphate dehydrogenase (GAPDH) as the reference genes. Primers for RT-PCR were designed based on sequences from the NCBI database using Primer Premier 5 software (Premier Biosoft International, PaloAlto, CA, USA). The primers were synthesized by Evrogen (Evrogen, Moscow, Russia), and are presented in the Supplementary Materials (Table S1).

The obtained data were processed using the Bio-Rad CFX Manager 2.1 and Microsoft Excel 2010 software packages. All the expression values were normalized relative to the Ct geometric mean of Actb and GAPDH genes. The delta-delta Ct method was used to evaluate expression.

### 4.6. Western Blotting

Western blotting was used to determine PSD95, NMDAR1, NMDAR2, GluR1, and GluR2 levels in mouse hippocampus. The animals were anesthetized with isoflurane, and the hippocampi were quickly removed, frozen in liquid nitrogen, and stored at −80 °C. Protein was extracted from the contralateral hippocampus for Western blot analysis based on prior immunohistochemistry results showing prominent changes in this region. The sample homogenization was performed using a homogenizing buffer consisting of 100 mM Tris, pH 7.4, 150 mM NaCl, 1 mM EGTA and 1 mM EDTA, 1% Triton X-100, and 0.5% sodium deoxycholate with a cocktail of protease inhibitors (cOmplete™, Sigma-Aldrich, St. Louis, Missouri, USA). The obtained homogenates were incubated on ice for 15 min, then centrifuged (16,000× *g*, 30 min, +4 °C). Supernatants were then collected. The final protein concentration was adjusted to 2 mg/mL. Samples were then diluted 1:1 with standard loading buffer (1× sample buffer, Biorad, Hercules, CA, USA) containing 5% 2-mercaptoethanol, placed in a water bath at 94.5 °C, and incubated for 5 min. Electrophoresis was performed using a Biorad system using Protean mini gel Any kDa gel cartridges (Biorad, Hercules, CA, USA) and Spectra Multicolor Broad Range Protein Ladder (Thermo Fisher Scientific, Waltham, MA, USA). The well loading was 60 μg of protein, and the current per gel was 15 mA with a constant voltage of 120 V. After electrophoresis, proteins were transferred to a PVDF membrane using a Turbo transblot transfer system (Biorad, Hercules, CA, USA). All transfer materials were used from the Transblot Turbo RTA Transfer Kit (Biorad). The membranes were then placed in blocking buffer (1× phosphate buffer containing 2% BSA, 0.1% Tween20, 0.05% Triton X-100) overnight. The next day, the blocking buffer was washed with PBS + 0.1% Tween20 and then incubated for 1 h with primary antibodies: anti-PSD-95 (1:1000, ab18258), anti-NMDAR1 (1:5000, ab109182), anti-NMDAR2 (1:1000, ab124913), anti-GluR1 (1:5000, ab183797 and anti-GluR2 (1:5000, ab206293), all from Abcam (Cambridge, UK). β-tubulin (1:500, ab6046) was used as a loading control. After incubation with primary antibodies, the membranes were washed again with PBS-T, then incubated for an hour with secondary antibodies Anti-Rabbit (Abcam) and Anti-Mouse (Abcam, Cambridge, UK). Western Blot ECL Substrate (Biorad, Hercules, CA, USA) was used to carry out the chemiluminescence reaction (1 mL of substrate per membrane, 5 min). Visualization was performed using the ChemiDoc gel documentation system (Biorad, Hercules, CA, USA). The resulting images were analyzed using the ImageLab 6.1 software package (Biorad, Hercules, CA, USA).

### 4.7. Statistical Analysis

Mean values ± standard error of the mean were used to present the study’s findings. The data’s normal distribution was ascertained using the Shapiro–Wilk test. Student’s *t*-test was used to assess the data. A significant threshold of *p* < 0.05 was established. All statistical tests were conducted using the Microsoft Excel program (Microsoft, Redmond, WA, USA).

## 5. Conclusions

This study demonstrates a clear link between neuropathic pain, neuroinflammation, and hippocampal dysfunction in mice. Specifically, the observed decrease in hippocampus-dependent functions, such as working spatial and long-term memory, alongside the increase in anxiety, appears to be a consequence of neuroinflammation within the hippocampus. This inflammation likely results in reduced expression of key synaptic components, including glutamate receptors and the postsynaptic scaffolding protein PSD-95. These molecular changes ultimately disrupt hippocampal neurogenesis, synaptic plasticity, and neuronal communication within the hippocampus, leading to the behavioral deficits observed. Therefore, this work identifies reductions in glutamate-receptor and PSD-95 expression as a critical mechanism underlying the detrimental impact of chronic pain on both cognitive and emotional processing. These results highlight the potential for therapeutic interventions targeting neuroinflammation to alleviate both the pain itself and the associated cognitive and affective comorbidities.

## Data Availability

The datasets supporting the finding of this study are available from the corresponding author upon reasonable request.

## Supplementary Materials

The following supporting information can be downloaded at: https://www.mdpi.com/article/10.3390/ijms26136460/s1.

## Abbreviations

The following abbreviations are used in this manuscript:

SNI	Spared Nerve Injury
CNS	Central Nervous System
LTP	Long-Term Potentiation
NMDA	N-Methyl-D-Aspartate
AMPA	α-Amino-3-hydroxy-5-methyl-4-isoxazolepropionic acid
PSD-95	Postsynaptic Density Protein 95
DG	Dentate Gyrus
CA1, CA3	Cornu Ammonis areas 1 and 3 of the hippocampus
GFAP	Glial Fibrillary Acidic Protein
Iba-1	Ionized Calcium-Binding Adapter Molecule 1
BIN1	Bridging Integrator 1
C3	Complement Component 3
CD68	Cluster of Differentiation 68
DCX	Doublecortin
Ki-67	Proliferation Marker Protein Ki-67
EPM	Elevated Plus Maze
OF	Open Field
NOR	Novel Object Recognition
RT-PCR	Real-Time Polymerase Chain Reaction
mRNA	Messenger Ribonucleic Acid
PBS	Phosphate Buffered Saline
DAB	Diaminobenzidine
BSA	Bovine Serum Albumin
SEM	Standard Error of the Mean
PVDF	Polyvinylidene Difluoride
CNS	Central Nervous System
ROS	Reactive Oxygen Species
ECL	Enhanced Chemiluminescence
