# Supplementary material for: Cognitive and Affective Dysregulation in Neuropathic Pain: Associated Hippocampal Remodeling and Microglial Activation
Source: Int J Mol Sci. 2025 Jul 4;26(13):6460. doi: 10.3390/ijms26136460 (PMC12249839; doi:10.3390/ijms26136460)
Supplement: Supplementary file 1 [file ijms-26-06460-s001.zip › Supplementary.pdf]

Name	Forward Seq	Reverse Seq
GAPDH	CTCCCACTCTTCCACCTTCG	GCCTCTCTTGCTCAGTGTCC
beta-Actin	GGCTGTATTCCCCTCCATCG	AGTCCTTCTGACCCATTCCCAC
CD68	CTCATCATTGGCCTGGTCCT	GTTGATTGTCGTCTGCGGG
PSD95	TCTGTGCGAGAGGTAGCAGA	AAGCACTCCGTGAACTCCTG
Bin1	ACTGAGTGGTGGCTGACAAG	TGAGTGCCAGAGAATCAGCG
GFAP	ATGTGTCCCCAGAGTTCTATTG	TTCACTACAAGAGCAGCCGTC
Component (C3)	GCCTCTCCTCTGACCTCTGG	AGTTCTTCGCACTGTTTCTGG

Table S1. The list of primers used in Real-time PCR.
