# Supplementary figures and images for: Cognitive and Affective Dysregulation in Neuropathic Pain: Associated Hippocampal Remodeling and Microglial Activation
Source: Int J Mol Sci. 2025 Jul 4;26(13):6460. doi: 10.3390/ijms26136460 (PMC12249839; doi:10.3390/ijms26136460)

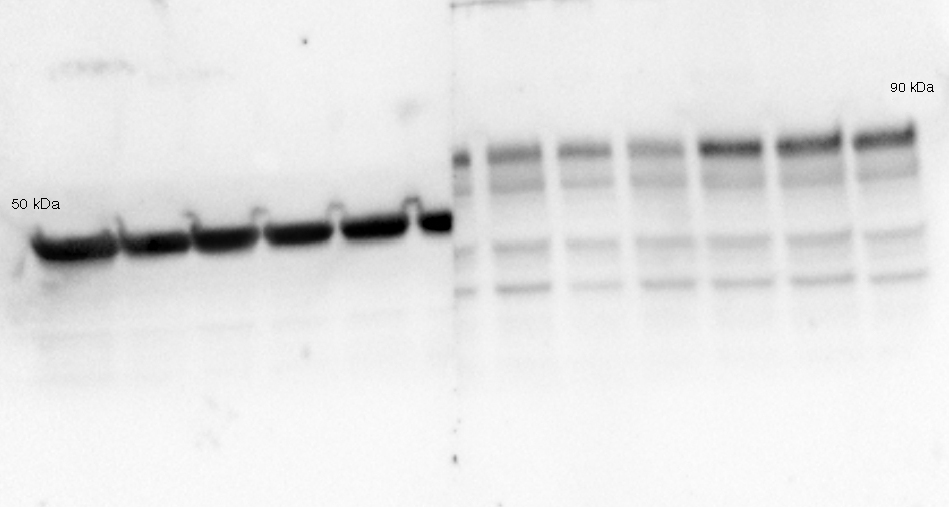

Supplement: Supplementary file 1 [file ijms-26-06460-s001.zip › Figure S1 Tubulin-PSD95.jpg]

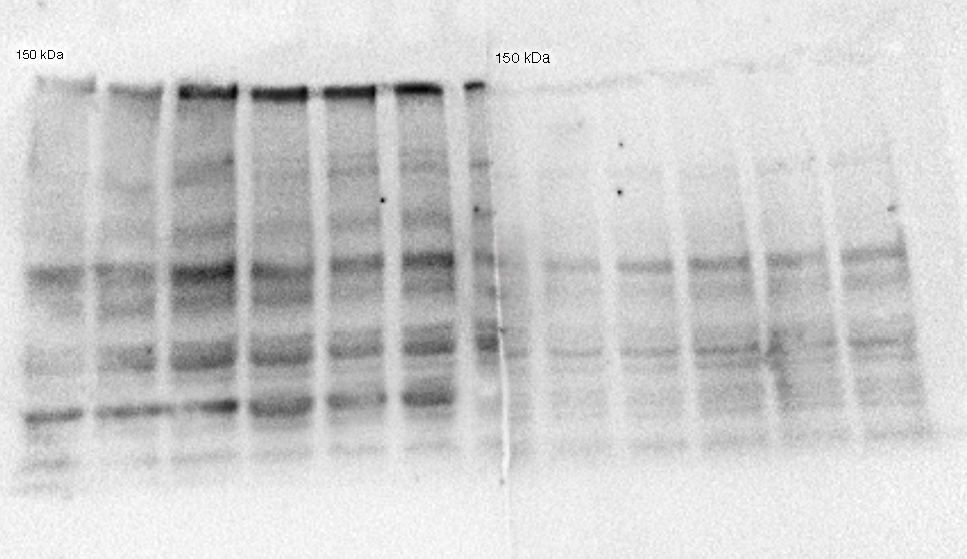

Supplement: Supplementary file 1 [file ijms-26-06460-s001.zip › Figure S2 NMDA2-NMDA1.jpg]

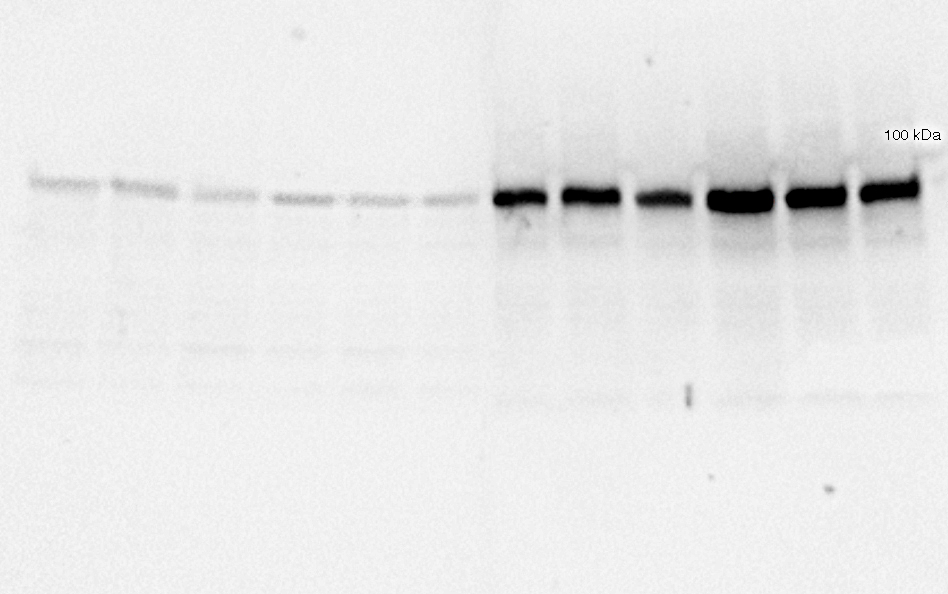

Supplement: Supplementary file 1 [file ijms-26-06460-s001.zip › Figure S3 GluR2-GluR1.jpg]
